# 1,1′,1′′,1′′′-(Oxydi­methane­tri­yl)tetra­kis­(4-fluoro­benzene)

**DOI:** 10.1107/S1600536814005984

**Published:** 2014-03-26

**Authors:** K. R. Roopashree, H. D. Kavitha, K. S. Katagi, O. Kotresh, H. C. Devarajegowda

**Affiliations:** a Department of Physics, Yuvaraja’s College (Constituent College), University of Mysore, Mysore 570005, Karnataka, India; b Department of Physics, Govt. Science College, Hassan 573 201, Karnataka, India; c Department of Chemistry, Karnatak Science College, Karnatak University, Dharwad, Karnataka 580001, India

## Abstract

In the title compound, C_26_H_18_F_4_O_2_, the dihedral angles between pairs of benzene rings linked to the same C atom are 80.55 (8) and 79.11 (7)°. The crystal packing features C—H⋯π inter­actions and shows stacking when viewed along the *c* axis.

## Related literature   

For biological applications of the benzhydryl ether unit, see: Brahmachari (2010 ▶); Weis *et al.* (2006 ▶); Van Der Zee & Hespe (1978 ▶); Nilsson *et al.* (1969 ▶); McGavack *et al.* (1948 ▶); Loew & Kaiser (1945 ▶); Pyo *et al.* (2004 ▶). For a related structure, see: Devarajegowda *et al.* (2011 ▶).
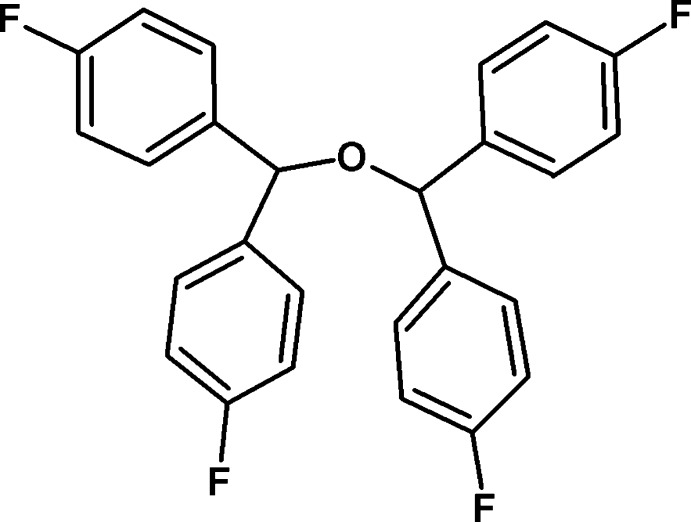


## Experimental   

### 

#### Crystal data  

C_26_H_18_F_4_O_2_
*M* *_r_* = 438.40
Triclinic, 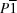

*a* = 8.1754 (2) Å
*b* = 8.9536 (2) Å
*c* = 15.3193 (4) Å
α = 104.965 (2)°
β = 95.175 (2)°
γ = 107.354 (2)°
*V* = 1016.87 (4) Å^3^
*Z* = 2
Mo *K*α radiation
μ = 0.11 mm^−1^
*T* = 296 K
0.24 × 0.20 × 0.12 mm

#### Data collection  

Bruker SMART CCD area-detector diffractometer
Absorption correction: ψ scan (*SADABS*; Sheldrick, 2007 ▶) *T*_min_ = 0.770, *T*_max_ = 1.000
24205 measured reflections
6598 independent reflections
4213 reflections with *I* > 2σ(*I*)
*R*_int_ = 0.030

#### Refinement  

*R*[*F* ^2^ > 2σ(*F* ^2^)] = 0.048
*wR*(*F* ^2^) = 0.132
*S* = 1.03
6598 reflections
280 parameters
H-atom parameters constrained
Δρ_max_ = 0.19 e Å^−3^
Δρ_min_ = −0.20 e Å^−3^

### 

Data collection: *SMART* (Bruker, 2001 ▶); cell refinement: *SAINT* (Bruker, 2001 ▶); data reduction: *SAINT*; program(s) used to solve structure: *SHELXS97* (Sheldrick, 2008 ▶); program(s) used to refine structure: *SHELXL97* (Sheldrick, 2008 ▶); molecular graphics: *ORTEP-3 for Windows* (Farrugia, 2012 ▶); software used to prepare material for publication: *SHELXL97*.

## Supplementary Material

Crystal structure: contains datablock(s) I, global. DOI: 10.1107/S1600536814005984/bv2231sup1.cif

Structure factors: contains datablock(s) I. DOI: 10.1107/S1600536814005984/bv2231Isup2.hkl

Supporting information file. DOI: 10.1107/S1600536814005984/bv2231Isup3.cml

CCDC reference: 992250

Additional supporting information:  crystallographic information; 3D view; checkCIF report

## Figures and Tables

**Table 1:** Hydrogen-bond geometry (Å, °) *Cg*4 is the centroid of the C26–C31 ring.

*D*—H⋯*A*	*D*—H	H⋯*A*	*D*⋯*A*	*D*—H⋯*A*
C7—H7⋯*Cg*4^i^	0.93	2.82	3.6834 (17)	154

Symmetry code: (i) 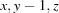.

**Fig. 1.**
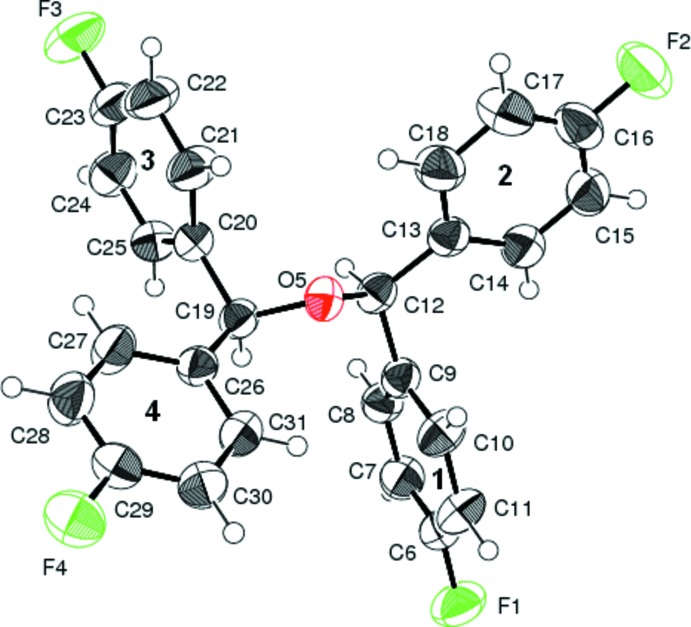
The molecular structure of the title compound. Displacement ellipsoids are drawn at the 50% probability level. Hydrogen atoms are shown as spheres of arbitrary radius.

**Fig. 2.**
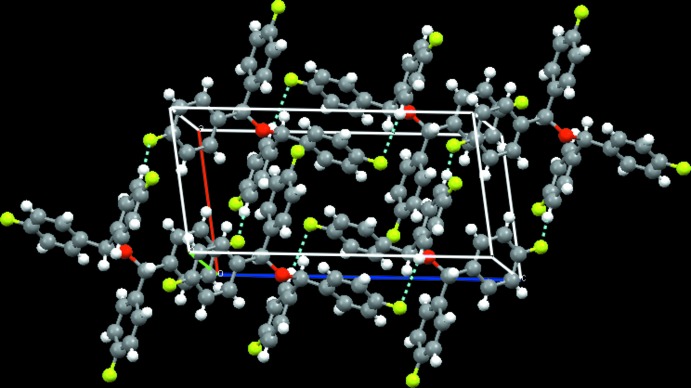
The packing of molecule of the title compound.

## supplementary crystallographic information

### 1. Comment

The benzhydryl ether moiety is abundant in a number of naturally occurring and biologically active compounds as well as molecules of potential clinical use and also exhibit various pharmacological potentials. In addition such molecules possess properties such as non-nucleoside reverse transcriptase inhibition (Brahmachari, 2010), anti-plasmodial and anti-trypanosomal action (Weis *et al.*, 2006), monoamine uptake inhibition, anti-depressant and anti-Parkinsonian activity (Van Der Zee & Hespe, 1978; Nilsson *et al.*, 1969), and anti-histaminic (McGavack *et al.*, 1948) and anti-spasmodic (Loew & Kaiser, 1945) action. Naturally occurring symmetrical bis(benzhydryl)ethers are also known to show promising therapeutic potential including significant anti-platelet aggregation efficacy (Pyo *et al.*, 2004). In this article we report the crystal structure of 1,1',1'',1^'''^-(oxydimethanetriyl)tetrakis (4-fluorobenzene)

The molecular unit of 1,1',1'',1^'''^-(oxydimethanetriyl)tetrakis (4-fluorobenzene) is shown in Fig. 1. The dihedral angles between each pair of benzene rings are 80.55 (8)° [(C6–C11) 1 and (C13–C18) 2] and 79.11 (7)° [(C20–C25) 3 and (C26–C31) 4] respectively. The crystal packing is stabilized by C7—H7···π Cg4[(C26–C31)] interactions and shows stacking when viewed along *c* axis. The bond distances and bond angles in the benzene ring system are in good agreement with those observed in related structures (Devarajegowda *et al.*, 2011).

### 2. Experimental

An oven-dried screw cap test tube was charged with a magnetic stir bar, benzhydrol (1 mmol), and p-toluenesulfonyl chloride (5 mol%). The tube was then evacuated and back-filled with nitrogen. The evacuation/ backfill sequence was repeated two additional times. The tube was placed in a preheated oil bath at 110°C, and the reaction mixture was stirred vigorously. The progress of the reaction was monitored by TLC, and on completion, the reaction mixture was cooled to room temperature. The reaction mixture was extracted with dried ethyl acetate (10 ml), and the extract was then concentrated under reduced pressure; the residue was purified *via* column chromatography using silica gel (60 to 120 mesh) and petrol ether-ethyl acetate mixture. white solid, 91% yield, m.p. 363 K.

### 3. Refinement

All H atoms were positioned geometrically, with C—H = 0.93 Å for aromatic H and refined using a riding model with *U*_iso_(H) = 1.2*U*_eq_(C) for H.

### Crystal data

**Table:**

C_26_H_18_F_4_O_2_	*Z* = 2
*M**_r_* = 438.40	*F*(000) = 452
Triclinic, *P*1	*D*_x_ = 1.432 Mg m^−^^3^
Hall symbol: -P 1	Melting point: 363 K
*a* = 8.1754 (2) Å	Mo *K*α radiation, λ = 0.71073 Å
*b* = 8.9536 (2) Å	Cell parameters from 3305 reflections
*c* = 15.3193 (4) Å	θ = 1.5–25.0°
α = 104.965 (2)°	µ = 0.11 mm^−^^1^
β = 95.175 (2)°	*T* = 296 K
γ = 107.354 (2)°	Plate, colourless
*V* = 1016.87 (4) Å^3^	0.24 × 0.20 × 0.12 mm

### Data collection

**Table:**

Bruker SMART CCD area-detector diffractometer	6598 independent reflections
Radiation source: fine-focus sealed tube	4213 reflections with *I* > 2σ(*I*)
Graphite monochromator	*R*_int_ = 0.030
ω and φ scans	θ_max_ = 31.3°, θ_min_ = 1.4°
Absorption correction: ψ scan (*SADABS*; Sheldrick, 2007)	*h* = −11→11
*T*_min_ = 0.770, *T*_max_ = 1.000	*k* = −12→13
24205 measured reflections	*l* = −22→22

### Refinement

**Table:**

Refinement on *F*^2^	Primary atom site location: structure-invariant direct methods
Least-squares matrix: full	Secondary atom site location: difference Fourier map
*R*[*F*^2^ > 2σ(*F*^2^)] = 0.048	Hydrogen site location: inferred from neighbouring sites
*wR*(*F*^2^) = 0.132	H-atom parameters constrained
*S* = 1.03	*w* = 1/[σ^2^(*F*_o_^2^) + (0.0526*P*)^2^ + 0.1212*P*] where *P* = (*F*_o_^2^ + 2*F*_c_^2^)/3
6598 reflections	(Δ/σ)_max_ < 0.001
280 parameters	Δρ_max_ = 0.19 e Å^−^^3^
0 restraints	Δρ_min_ = −0.20 e Å^−^^3^

### Special details

**Table:**

Experimental. IR (νmax, KBr) cm^-1^: 3,069, 3,057, 2,925, 1,603, 1,507, 1,422, 1,408, 1,298, 1,225, 1,178, 1,155, 1,101, 1,029, 859, 837, 818. ^1^H NMR (CDCl_3_, 400 MHz, δ): 7.19 to 7.16 (m, 8H, Ar H), 6.94 to 6.88 (m, 8H, Ar H), 5.22 (s, 2H, CH). 13 C NMR (CDCl_3_, 100 MHz, δ): 163.52, 161.07, 137.51, 137.48, 128.82, 128.74, 115.59, 115.38, 78.91. TOF-MS: 445.98 ([*M*^+^ Na]^+^). Anal. found: C, 73.89; H, 4.28. C_26_H_18_F_4_O requires C, 73.93; H, 4.30%
Geometry. All s.u.'s (except the s.u. in the dihedral angle between two l.s. planes) are estimated using the full covariance matrix. The cell s.u.'s are taken into account individually in the estimation of s.u.'s in distances, angles and torsion angles; correlations between s.u.'s in cell parameters are only used when they are defined by crystal symmetry. An approximate (isotropic) treatment of cell s.u.'s is used for estimating s.u.'s involving l.s. planes.
Refinement. Refinement of *F*^2^ against ALL reflections. The weighted *R*-factor *wR* and goodness of fit *S* are based on *F*^2^, conventional *R*-factors *R* are based on *F*, with *F* set to zero for negative *F*^2^. The threshold expression of *F*^2^ > 2σ(*F*^2^) is used only for calculating *R*-factors(gt) *etc*. and is not relevant to the choice of reflections for refinement. *R*-factors based on *F*^2^ are statistically about twice as large as those based on *F*, and *R*- factors based on ALL data will be even larger.

### Fractional atomic coordinates and isotropic or equivalent isotropic displacement parameters (Å^2^)

**Table:**

	*x*	*y*	*z*	*U*_iso_*/*U*_eq_	
F1	0.51662 (13)	−0.15696 (11)	0.07690 (7)	0.0851 (3)	
F3	1.71199 (12)	0.98406 (12)	0.40107 (7)	0.0887 (3)	
F2	0.68830 (14)	0.68143 (15)	0.64449 (6)	0.0910 (3)	
F4	0.80529 (13)	0.76746 (12)	−0.09958 (6)	0.0729 (3)	
O5	0.91588 (11)	0.58730 (10)	0.26211 (6)	0.0450 (2)	
C6	0.61205 (19)	−0.00592 (16)	0.13522 (10)	0.0564 (3)	
C7	0.77809 (19)	0.01998 (17)	0.17440 (10)	0.0575 (3)	
H7	0.8257	−0.0639	0.1627	0.069*	
C8	0.87448 (17)	0.17561 (16)	0.23243 (9)	0.0495 (3)	
H8	0.9887	0.1962	0.2594	0.059*	
C9	0.80425 (16)	0.29983 (14)	0.25068 (8)	0.0437 (3)	
C10	0.63362 (17)	0.26622 (16)	0.20986 (10)	0.0566 (3)	
H10	0.5835	0.3483	0.2224	0.068*	
C11	0.53683 (18)	0.11302 (17)	0.15099 (11)	0.0611 (4)	
H11	0.4232	0.0916	0.1228	0.073*	
C12	0.91059 (16)	0.47045 (14)	0.31180 (8)	0.0437 (3)	
H12	1.0300	0.4726	0.3286	0.052*	
C13	0.84427 (16)	0.52578 (15)	0.39945 (8)	0.0458 (3)	
C14	0.75351 (19)	0.41433 (18)	0.44062 (10)	0.0581 (3)	
H14	0.7276	0.3027	0.4123	0.070*	
C15	0.7006 (2)	0.4656 (2)	0.52311 (10)	0.0665 (4)	
H15	0.6397	0.3900	0.5505	0.080*	
C16	0.7398 (2)	0.6293 (2)	0.56319 (10)	0.0644 (4)	
C17	0.8300 (2)	0.7431 (2)	0.52598 (11)	0.0736 (5)	
H17	0.8562	0.8543	0.5553	0.088*	
C18	0.8823 (2)	0.69075 (18)	0.44369 (10)	0.0637 (4)	
H18	0.9442	0.7679	0.4175	0.076*	
C19	1.04230 (15)	0.59122 (14)	0.20239 (8)	0.0436 (3)	
H19	1.0426	0.4790	0.1771	0.052*	
C20	1.22295 (16)	0.69598 (14)	0.25586 (8)	0.0446 (3)	
C21	1.25308 (19)	0.85361 (16)	0.31264 (11)	0.0630 (4)	
H21	1.1613	0.8948	0.3175	0.076*	
C22	1.4173 (2)	0.95001 (18)	0.36192 (11)	0.0695 (4)	
H22	1.4368	1.0549	0.4006	0.083*	
C23	1.54990 (19)	0.88794 (18)	0.35260 (10)	0.0607 (4)	
C24	1.52757 (19)	0.73525 (19)	0.29736 (10)	0.0610 (4)	
H24	1.6210	0.6965	0.2918	0.073*	
C25	1.36120 (17)	0.63864 (16)	0.24939 (9)	0.0513 (3)	
H25	1.3429	0.5330	0.2121	0.062*	
C26	0.98039 (16)	0.64536 (14)	0.12351 (8)	0.0435 (3)	
C27	1.09134 (18)	0.75717 (18)	0.08976 (10)	0.0576 (3)	
H27	1.2077	0.8060	0.1183	0.069*	
C28	1.0337 (2)	0.79841 (18)	0.01455 (11)	0.0629 (4)	
H28	1.1099	0.8735	−0.0078	0.076*	
C29	0.86398 (19)	0.72695 (16)	−0.02568 (9)	0.0521 (3)	
C30	0.74903 (19)	0.6152 (2)	0.00409 (10)	0.0636 (4)	
H30	0.6331	0.5672	−0.0252	0.076*	
C31	0.80857 (18)	0.57483 (19)	0.07884 (10)	0.0597 (4)	
H31	0.7313	0.4982	0.0998	0.072*	

### Atomic displacement parameters (Å^2^)

**Table:**

	*U*^11^	*U*^22^	*U*^33^	*U*^12^	*U*^13^	*U*^23^
F1	0.0678 (6)	0.0598 (5)	0.0926 (7)	0.0021 (4)	0.0069 (5)	−0.0112 (5)
F3	0.0592 (6)	0.0754 (6)	0.1019 (8)	−0.0058 (5)	−0.0223 (5)	0.0227 (5)
F2	0.0886 (7)	0.1324 (9)	0.0540 (5)	0.0475 (7)	0.0217 (5)	0.0158 (6)
F4	0.0851 (6)	0.0804 (6)	0.0610 (5)	0.0351 (5)	0.0057 (5)	0.0285 (5)
O5	0.0444 (5)	0.0434 (4)	0.0515 (5)	0.0155 (4)	0.0136 (4)	0.0186 (4)
C6	0.0527 (8)	0.0478 (7)	0.0547 (8)	0.0039 (6)	0.0114 (6)	0.0066 (6)
C7	0.0586 (8)	0.0500 (7)	0.0621 (8)	0.0206 (6)	0.0145 (7)	0.0096 (6)
C8	0.0451 (7)	0.0522 (7)	0.0503 (7)	0.0174 (6)	0.0061 (5)	0.0133 (6)
C9	0.0419 (6)	0.0438 (6)	0.0452 (6)	0.0117 (5)	0.0066 (5)	0.0165 (5)
C10	0.0443 (7)	0.0476 (7)	0.0758 (9)	0.0143 (6)	0.0032 (6)	0.0188 (6)
C11	0.0416 (7)	0.0569 (8)	0.0737 (9)	0.0063 (6)	−0.0016 (6)	0.0168 (7)
C12	0.0388 (6)	0.0433 (6)	0.0494 (7)	0.0127 (5)	0.0046 (5)	0.0168 (5)
C13	0.0398 (6)	0.0506 (6)	0.0449 (6)	0.0135 (5)	0.0006 (5)	0.0148 (5)
C14	0.0568 (8)	0.0578 (8)	0.0535 (8)	0.0103 (6)	0.0080 (6)	0.0177 (6)
C15	0.0572 (9)	0.0849 (11)	0.0518 (8)	0.0115 (8)	0.0092 (7)	0.0254 (8)
C16	0.0565 (8)	0.0952 (12)	0.0429 (7)	0.0334 (8)	0.0055 (6)	0.0146 (8)
C17	0.0945 (13)	0.0672 (9)	0.0567 (9)	0.0327 (9)	0.0128 (9)	0.0082 (8)
C18	0.0780 (10)	0.0537 (8)	0.0557 (8)	0.0183 (7)	0.0131 (7)	0.0142 (6)
C19	0.0405 (6)	0.0379 (5)	0.0487 (6)	0.0104 (5)	0.0100 (5)	0.0096 (5)
C20	0.0437 (6)	0.0411 (6)	0.0448 (6)	0.0094 (5)	0.0080 (5)	0.0116 (5)
C21	0.0510 (8)	0.0473 (7)	0.0777 (10)	0.0123 (6)	0.0082 (7)	0.0027 (7)
C22	0.0633 (9)	0.0460 (7)	0.0765 (10)	0.0033 (7)	0.0021 (8)	0.0012 (7)
C23	0.0491 (8)	0.0570 (8)	0.0626 (9)	0.0000 (6)	−0.0056 (6)	0.0215 (7)
C24	0.0471 (7)	0.0660 (9)	0.0685 (9)	0.0181 (7)	0.0011 (6)	0.0220 (7)
C25	0.0498 (7)	0.0501 (7)	0.0509 (7)	0.0160 (6)	0.0042 (6)	0.0122 (6)
C26	0.0427 (6)	0.0386 (5)	0.0448 (6)	0.0114 (5)	0.0095 (5)	0.0071 (5)
C27	0.0436 (7)	0.0597 (8)	0.0660 (9)	0.0070 (6)	0.0060 (6)	0.0265 (7)
C28	0.0601 (9)	0.0610 (8)	0.0680 (9)	0.0104 (7)	0.0120 (7)	0.0312 (7)
C29	0.0614 (8)	0.0532 (7)	0.0445 (7)	0.0257 (6)	0.0091 (6)	0.0122 (6)
C30	0.0477 (8)	0.0796 (10)	0.0537 (8)	0.0110 (7)	0.0023 (6)	0.0181 (7)
C31	0.0473 (7)	0.0656 (8)	0.0540 (8)	0.0008 (6)	0.0055 (6)	0.0202 (7)

### Geometric parameters (Å, º)

**Table:**

F1—C6	1.3626 (15)	C17—C18	1.379 (2)
F3—C23	1.3639 (16)	C17—H17	0.9300
F2—C16	1.3628 (16)	C18—H18	0.9300
F4—C29	1.3642 (16)	C19—C26	1.5131 (18)
O5—C12	1.4375 (14)	C19—C20	1.5135 (16)
O5—C19	1.4395 (14)	C19—H19	0.9800
C6—C7	1.360 (2)	C20—C25	1.3751 (19)
C6—C11	1.362 (2)	C20—C21	1.3890 (18)
C7—C8	1.3898 (18)	C21—C22	1.381 (2)
C7—H7	0.9300	C21—H21	0.9300
C8—C9	1.3763 (18)	C22—C23	1.361 (2)
C8—H8	0.9300	C22—H22	0.9300
C9—C10	1.3865 (18)	C23—C24	1.358 (2)
C9—C12	1.5128 (16)	C24—C25	1.3885 (19)
C10—C11	1.3806 (19)	C24—H24	0.9300
C10—H10	0.9300	C25—H25	0.9300
C11—H11	0.9300	C26—C27	1.3795 (18)
C12—C13	1.5134 (17)	C26—C31	1.3837 (18)
C12—H12	0.9800	C27—C28	1.382 (2)
C13—C18	1.3816 (19)	C27—H27	0.9300
C13—C14	1.3827 (18)	C28—C29	1.352 (2)
C14—C15	1.383 (2)	C28—H28	0.9300
C14—H14	0.9300	C29—C30	1.358 (2)
C15—C16	1.360 (2)	C30—C31	1.378 (2)
C15—H15	0.9300	C30—H30	0.9300
C16—C17	1.354 (2)	C31—H31	0.9300
			
C12—O5—C19	112.25 (9)	O5—C19—C26	107.41 (10)
C7—C6—C11	122.94 (13)	O5—C19—C20	110.56 (9)
C7—C6—F1	118.79 (13)	C26—C19—C20	114.95 (10)
C11—C6—F1	118.27 (13)	O5—C19—H19	107.9
C6—C7—C8	117.98 (13)	C26—C19—H19	107.9
C6—C7—H7	121.0	C20—C19—H19	107.9
C8—C7—H7	121.0	C25—C20—C21	118.30 (12)
C9—C8—C7	121.28 (13)	C25—C20—C19	121.22 (11)
C9—C8—H8	119.4	C21—C20—C19	120.48 (12)
C7—C8—H8	119.4	C22—C21—C20	120.93 (14)
C8—C9—C10	118.37 (12)	C22—C21—H21	119.5
C8—C9—C12	121.32 (11)	C20—C21—H21	119.5
C10—C9—C12	120.29 (11)	C23—C22—C21	118.46 (14)
C11—C10—C9	121.11 (13)	C23—C22—H22	120.8
C11—C10—H10	119.4	C21—C22—H22	120.8
C9—C10—H10	119.4	C24—C23—C22	122.88 (13)
C6—C11—C10	118.30 (13)	C24—C23—F3	118.94 (15)
C6—C11—H11	120.9	C22—C23—F3	118.19 (14)
C10—C11—H11	120.9	C23—C24—C25	118.05 (14)
O5—C12—C9	109.77 (9)	C23—C24—H24	121.0
O5—C12—C13	108.00 (9)	C25—C24—H24	121.0
C9—C12—C13	114.58 (10)	C20—C25—C24	121.37 (13)
O5—C12—H12	108.1	C20—C25—H25	119.3
C9—C12—H12	108.1	C24—C25—H25	119.3
C13—C12—H12	108.1	C27—C26—C31	117.42 (13)
C18—C13—C14	117.97 (13)	C27—C26—C19	122.56 (11)
C18—C13—C12	120.56 (12)	C31—C26—C19	119.91 (11)
C14—C13—C12	121.36 (12)	C26—C27—C28	121.57 (13)
C13—C14—C15	121.28 (14)	C26—C27—H27	119.2
C13—C14—H14	119.4	C28—C27—H27	119.2
C15—C14—H14	119.4	C29—C28—C27	118.40 (13)
C16—C15—C14	118.31 (14)	C29—C28—H28	120.8
C16—C15—H15	120.8	C27—C28—H28	120.8
C14—C15—H15	120.8	C28—C29—C30	122.66 (13)
C17—C16—C15	122.50 (14)	C28—C29—F4	118.94 (13)
C17—C16—F2	118.62 (16)	C30—C29—F4	118.40 (13)
C15—C16—F2	118.88 (15)	C29—C30—C31	118.27 (13)
C16—C17—C18	118.76 (15)	C29—C30—H30	120.9
C16—C17—H17	120.6	C31—C30—H30	120.9
C18—C17—H17	120.6	C30—C31—C26	121.68 (13)
C17—C18—C13	121.17 (15)	C30—C31—H31	119.2
C17—C18—H18	119.4	C26—C31—H31	119.2
C13—C18—H18	119.4		
			
C11—C6—C7—C8	−0.5 (2)	C12—O5—C19—C26	152.38 (9)
F1—C6—C7—C8	178.88 (12)	C12—O5—C19—C20	−81.48 (11)
C6—C7—C8—C9	0.6 (2)	O5—C19—C20—C25	129.54 (12)
C7—C8—C9—C10	0.2 (2)	C26—C19—C20—C25	−108.67 (13)
C7—C8—C9—C12	−178.35 (12)	O5—C19—C20—C21	−50.75 (16)
C8—C9—C10—C11	−1.1 (2)	C26—C19—C20—C21	71.04 (15)
C12—C9—C10—C11	177.40 (13)	C25—C20—C21—C22	−0.5 (2)
C7—C6—C11—C10	−0.4 (2)	C19—C20—C21—C22	179.81 (14)
F1—C6—C11—C10	−179.81 (13)	C20—C21—C22—C23	0.9 (3)
C9—C10—C11—C6	1.3 (2)	C21—C22—C23—C24	−0.3 (3)
C19—O5—C12—C9	−80.08 (11)	C21—C22—C23—F3	179.66 (14)
C19—O5—C12—C13	154.36 (9)	C22—C23—C24—C25	−0.7 (2)
C8—C9—C12—O5	122.12 (12)	F3—C23—C24—C25	179.29 (13)
C10—C9—C12—O5	−56.39 (15)	C21—C20—C25—C24	−0.6 (2)
C8—C9—C12—C13	−116.18 (13)	C19—C20—C25—C24	179.09 (12)
C10—C9—C12—C13	65.31 (15)	C23—C24—C25—C20	1.2 (2)
O5—C12—C13—C18	−32.95 (16)	O5—C19—C26—C27	137.23 (12)
C9—C12—C13—C18	−155.61 (13)	C20—C19—C26—C27	13.75 (17)
O5—C12—C13—C14	151.03 (12)	O5—C19—C26—C31	−46.67 (15)
C9—C12—C13—C14	28.37 (16)	C20—C19—C26—C31	−170.15 (12)
C18—C13—C14—C15	0.6 (2)	C31—C26—C27—C28	0.4 (2)
C12—C13—C14—C15	176.73 (13)	C19—C26—C27—C28	176.56 (13)
C13—C14—C15—C16	0.1 (2)	C26—C27—C28—C29	0.4 (2)
C14—C15—C16—C17	−0.8 (2)	C27—C28—C29—C30	−0.9 (2)
C14—C15—C16—F2	179.99 (13)	C27—C28—C29—F4	179.57 (13)
C15—C16—C17—C18	0.8 (3)	C28—C29—C30—C31	0.6 (2)
F2—C16—C17—C18	−179.99 (14)	F4—C29—C30—C31	−179.86 (13)
C16—C17—C18—C13	−0.1 (3)	C29—C30—C31—C26	0.2 (2)
C14—C13—C18—C17	−0.6 (2)	C27—C26—C31—C30	−0.7 (2)
C12—C13—C18—C17	−176.76 (14)	C19—C26—C31—C30	−176.97 (13)

### Hydrogen-bond geometry (Å, º)

Cg4 is the centroid of the C26–C31 ring.

**Table:**

*D*—H···*A*	*D*—H	H···*A*	*D*···*A*	*D*—H···*A*
C7—H7···*Cg*4^i^	0.93	2.82	3.6834 (17)	154

Symmetry code: (i) *x*, *y*−1, *z*.
